# *ELOVL2*, *PRKG2*, and *EDARADD* DNA Methylation Strongly Estimate Indonesian Adolescents

**DOI:** 10.3390/diagnostics14161767

**Published:** 2024-08-14

**Authors:** Nurtami Soedarsono, Muhammad Garry Syahrizal Hanafi, Bambang Tri Hartomo, Elza Ibrahim Auerkari

**Affiliations:** 1 Division of Forensic Odontology, Department of Oral Biology, Faculty of Dentistry, Universitas Indonesia, Kota Depok, DKI, Jakarta 10430, Indonesia; muhammad.garry@ui.ac.id (M.G.S.H.); elza.ibrahim@ui.ac.id (E.I.A.); 2 Department of Dental Medicine, Faculty of Medicine, Universitas Jenderal Soedirman, Purwokerto 53122, Indonesia; bambang.tri.hartomo@gmail.com

**Keywords:** adolescent, age estimation, DNA methylation, epigenetic, forensic identification

## Abstract

Recently, there has been a growing interest in using DNA methylation analysis for age estimation. Despite this growing interest, there is a scarcity of research on the potential of DNA methylation as a biomarker for age estimation in Indonesia. This study aims to investigate the applicability of *ELOVL2*, *PRKG2*, and *EDARADD* genes for forensic identification in the 11–20 age group among Indonesians. This research utilizes 43 archived blood samples from healthy individuals who underwent blood tests at the Gatot Soebroto Army Hospital (RSPAD) in Central Jakarta, Indonesia. The methylation-specific PCR (MSP) technique assessed the DNA methylation level. The key findings of this study include (1) a strong positive correlation between methylation levels in the *ELOVL2* gene and age; (2) a strong negative correlation between methylation levels in *PRKG2* and *EDARADD* genes with age; (3) the development of three linear regression formulas for age prediction; and (4) mean absolute error (MAE) values derived from this research, which are ±0.48 for *ELOVL2* gene regression formula, ±0.58 for *PRKG2* gene regression formula, and ±0.72 for *EDARADD* gene regression formula. In summary, this study explores the potential of DNA methylation analysis for age estimation in Indonesia, focusing on *ELOVL2*, *PRKG2*, and *EDARADD* genes in the 11–20 age group. The findings underscore the applicability of DNA methylation analysis in forensic identification and age estimation, paving the way for future research in this field.

## 1. Introduction

Age estimation is crucial to forensic, legal, and anthropological investigations [1]. Age estimations can be used for both living and deceased individuals. When dealing with living individuals, situations that require age estimation occur in various contexts, such as marriage, determining the age of athletes in a competition, issuing identity cards and other essential papers, school enrollment, child adoption, immigration hearings, and judicial proceedings [2,3]. In such cases, it is necessary to verify the chronological age on official documents using scientific methods to estimate the biological age (the actual age). This verification is essential to prevent occurrences of age falsification [4,5,6]. In Indonesia, age verification regulations primarily cover individuals between the ages of 8 and 21. Thus, adolescents between the ages of 11 and 20 in Indonesia are especially prone to falsifying their age because of the numerous laws and important papers issued within this age range [7].

Morphological or radiological techniques are the most reliable method for estimating age. However, these methods still have limitations. These include the increased subjectivity that comes with observer judgments, the absence of a universally applicable atlas that can account for different races and populations, and the limited effectiveness of age range observations—which are mainly only helpful for individuals in the growth age range, and not practical when dealing with samples consisting only of biological materials [8,9,10].

A promising approach to enhance age estimation methodologies entails biomolecular analysis, specifically through DNA methylation study [11,12]. DNA methylation analysis is considered more objective and thus can reduce researchers’ subjective judgments. Furthermore, the technique does not require an atlas for guiding, making it potentially usable in all populations worldwide. Another advantage of using this method is that it can be used in a wide range of age observations, including assessments of individuals in old age, which often cannot be done through an odontological or anthropological approach. Moreover, using this method with limited biological material is also possible, making it suitable for post-mortem tests that involve only minor body parts [13,14,15,16].

Presently, a significant number of CpG sites that show relationships with age have been found for methylation study. Multiple genes have consistently demonstrated substantial connections with aging [10,13,17,18,19,20]. However, most of these studies focus on adults (individuals aged 18 and older), which leads to a lack of in-depth analysis explicitly examining DNA methylation patterns during the early stages of life, including adolescents. It is hypothesized that individuals in this age range undergo more significant changes in methylation patterns than adults. This theory arises from the increased activation of the immune system and the rapid periods of growth and development during adolescence. In addition, it is considered that environmental factors have a more significant influence on methylation patterns within this particular age range [16,21].

As a result, several researchers have focused on comprehending the patterns of DNA methylation during the early stages of life [16,21,22,23,24,25,26]. The researchers focused primarily on CpG sites inside specific gene markers that showed the strongest associations in the adolescent age group. For instance, Alisch et al. examined methylation at 27,578 CpG sites. They found that 2078 specific locations were strongly associated with age progression in individuals between the ages of 3 and 17 [21]. Expanding on the studies, Freire-Aradas et al. undertook further investigations to identify gene markers that showed the strongest correlation with changes in methylation patterns at an early age. The study found that *ELOVL2*, *PRKG2*, and *EDARADD* were among the most potent genes linked to young age [16]. In light of the background above, this study aims to examine the correlation among DNA methylation levels in the *ELOVL2*, *PRKG2*, and *EDARADD* genes and the age group of 11 to 20 years in the Indonesian population.

## 2. Materials and Methods

### 2.1. Sampling Method

The sampling was conducted in the Clinical Pathology Laboratory of the Gatot Subroto Army Central Hospital (RSPAD) in Central Jakarta, Indonesia. Afterward, the storage and extraction of DNA samples were carried out at the Oral Biology Laboratory, Faculty of Dentistry, Universitas Indonesia (FKG UI), and the analysis of DNA methylation was conducted at the Human Reproductive, Infertility and Family Planning IMERI, Faculty of Medicine, Universitas Indonesia.

The study focused on the Indonesian population, using stored blood samples from individuals aged 11–20 who had sought medical treatment and met the inclusion criteria. The compilation of stored blood samples complied with the ethical principles established by the FKG UI and RSPAD, specifically in addressing the difficulties presented by the COVID-19 pandemic.

The inclusion criteria consisted of samples from Indonesians between the ages of 11 and 20, as confirmed by medical records and Indonesian ID Cards. On the other hand, the exclusion criteria included samples from individuals who had a past of age-related degenerative diseases (such as type II diabetes mellitus, Hutchinson–Gilford Disease, cardiovascular disorders, Prader–Willi Syndrome, Angelman Syndrome, Beckwith–Wiedemann Syndrome, Werner Syndrome, glioblastoma multiforme, acute myeloid leukemia, etc.), individuals who had not been tested for Sars-CoV-2 using the RT-PCR method, and individuals who tested positive for Sars-CoV-2 using the RT-PCR method.

We filtered approximately 45,000 blood samples from the archives, yielding only 54 samples that satisfied the inclusion criteria. Out of these, upon further analysis of medical records, six samples had systemic disorders, specifically systemic lupus erythematosus in three samples, type 1 diabetes mellitus in one sample, and thalassemia in two samples. In addition, five samples had a low concentration of DNA after isolation, making them unsuitable for further analysis in the bisulfite and methylation testing stages. As a result, these samples were excluded from the investigation. Therefore, 43 samples were deemed suitable for bisulfite treatment and DNA methylation analysis.

### 2.2. Bisulfite Conversion Testing

The Bisulfite Conversion Testing employed the Qiagen EpiTect Bisulfite kit (Hilden, Germany), capable of processing DNA quantities ranging from 1 ng to 2 µg in a 20 µL volume. The Bisulfite Mix aliquot was suitable for eight conversion reactions. The DNA Protect Buffer color change from green to blue indicated proper mixing and pH for the bisulfite conversion reaction. All centrifugation steps were performed at room temperature (15–25 °C).

Preparations included adding 30 mL of ethanol (96–100%) to BW Buffer and storing it at room temperature (15–25 °C). Similarly, 27 mL of ethanol (96–100%) was added to Buffer BD and stored at 2–8 °C.

For RNA carrier preparation, 310 μL of RNAse-free water was added to a lyophilized RNA carrier (310 μg) to achieve a 1 μg/μL solution. Subsequently, an RNA carrier was added to Buffer BL, which is necessary for DNA binding in low-target-molecule scenarios. Buffer BL precipitate was dissolved by heating (maximum 70 °C) with gentle agitation.

### 2.3. DNA Methylation Examination by MSP Method

DNA methylation analysis was performed using MSP with the Toyobo KOD Multi and Epi kit (Osaka, Japan). Specific primers for the *ELOVL2*, *PRKG2*, and *EDARADD* genes were used to differentiate between methylated (M) and unmethylated (U) cytosines.

A Qiagen EpiTect PCR Control Kit (Hilden, Germany) served as methylated and unmethylated control DNA. The primers for MSP were designed using MethPrimer and NCBI Genome. Initial reference sequences for *ELOVL2* primers were taken from a study by Hartomo et al. [27], while *PRKG2* and *EDARADD* were determined based on sequences from Freire-Aradas et al. [16].

Verification of gene presence was conducted using the UCSC Genome Browser, and the genome locations were confirmed before designing the primers. Primers were checked for secondary structure using Oligoanalyzer IDT to avoid performance issues. The final primer sequences used are listed in Table 1 below.

### 2.4. Electrophoresis and ImageJ

**Figure 1:**
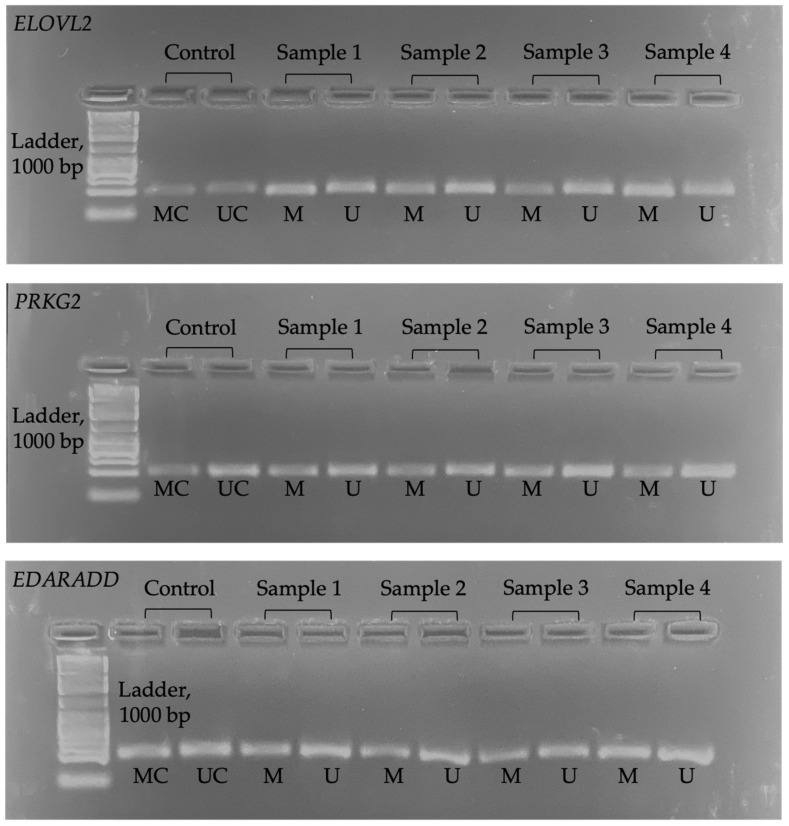
Electrophoresis image for all genes. MC = methylated control; UC = unmethylated control; M = methylated; U = unmethylated.

**Figure 2:**
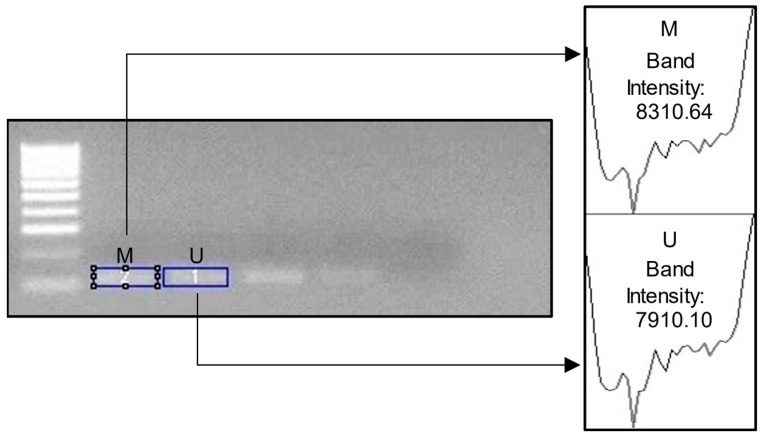
Example image of ImageJ usage. M = methylated; U = unmethylated. For the percentage of DNA methylation, we calculated the following: M Band Intensity/Total Intensity (M + U) × 100. In the example above, the calculation would be 83.1064/16.22074 × 100= 51.23%.

We used ImageJ (version 1.5.3) software to determine the pixel intensity in the band of the electrophoresis image (JPG format). The opacity of the electrophoresis image shows a higher methylation level. Figure 1 shows the electrophoresis images for each gene. Meanwhile, Figure 2 shows an example of ImageJ calculation for methylation percentage.

### 2.5. Statistical Analysis

We used two observers to observe DNA methylation intensity for each gene that was quantified by analyzing electrophoresis band patterns using ImageJ software. Descriptive statistics were calculated for research subjects, including mean, standard error of the mean, median, mode, minimum, and maximum values.

The Kappa test was used to assess the agreement between two observers. Data normality was assessed using the Shapiro–Wilk test. Given the non-normal distribution, the Kruskal–Wallis test was employed, followed by the Mann–Whitney test as a post-hoc analysis to determine specific differences between methylation levels.

The correlation between gene methylation and age was analyzed using the Spearman test, and scatter plots were created to illustrate these relationships. Linear regression tests were conducted to develop a multivariate regression formula for age prediction. The predictive accuracy of the regression formula was evaluated using mean absolute error (MAE), R squared (R^2^), and root mean squared error (RMSE) analysis with a confidence interval (CI) of 95%. In addition, we performed a multicollinearity test (tolerance and variance inflation factor [VIF]) to verify that the independent variables in the regression models are not strongly correlated linearly. We also performed a power analysis to ascertain whether the sample size is sufficient to detect a significant effect, assuming such an effect exists. All statistical analyses were performed using SPSS (version 27) and Microsoft Excel (version 16.86).

## 3. Results

### 3.1. Samples Characteristics

The age range in this study was 11.7–20.9 years, with a mean age of 17.6 years (SD, ±2.6) (Table 2). The highest frequency was age 20 years with 11 samples (25.6%), and the lowest was age 11 years with 1 sample (2.3%). The frequency of sex was dominated by males, with 28 samples (65.1%), while the frequency of female samples was 15 (34.9%).

### 3.2. DNA Methylation Status

Before observing the DNA methylation level by two observers, we conducted a common perception of the variables observed in this study. We standardized the use of ImageJ software by using the same observation protocol. Then, we used Kappa analysis to measure the level of agreement or consistency between two observers. Table 3 shows the Kappa test result. The agreement value showed 0.909, with the indicator close to 1. In addition, the significance of the agreement between Observer 1 and Observer 2 was 0.000. Because *p* < 0.05 means there is a significant agreement between observers, it can be concluded that Observer 1 and Observer 2 are consistent.

**Figure 3:**
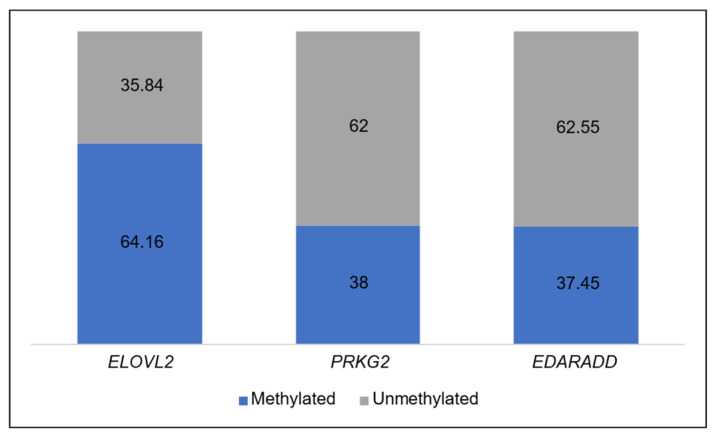
Average percentage level of methylated and unmethylated in each gene.

The average methylation levels among the three genes vary from one to another. From Table 4, it can be observed that *ELOVL2* has methylation levels above 50%. Meanwhile, the other two genes (*PRKG2* and *EDARADD*) have methylation levels around 30%. Figure 3 provides a visual representation of the methylation level status for each gene. The blue color represents the methylation level, while the grey color represents the unmethylation level.

After conducting a normality test on the data using the Shapiro–Wilk analysis, the data distribution that was found for methylation levels in each gene was not expected. Therefore, the Kruskal–Wallis analysis was chosen to determine whether there were DNA methylation level differences among genes. In Table 5, a *p*-value of <0.001 was obtained, indicating a statistically significant difference. Post-hoc analysis following the Kruskal–Wallis test, specifically Mann–Whitney analysis, was performed to identify which genes exhibit significant differences. From Table 6, it can be observed that there are significant differences in almost all genes (*p* < 0.05), except between *PRKG2* and *EDARADD*, which have a *p*-value > 0.05.

### 3.3. Correlation Analysis

**Figure 4:**
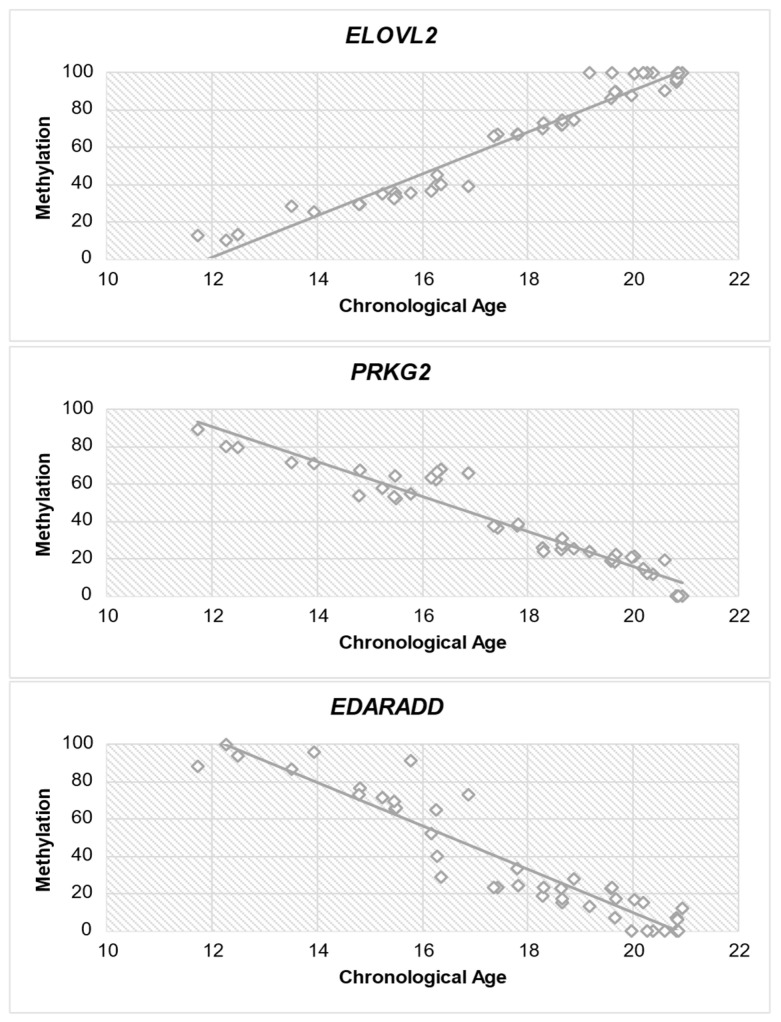
Diagram of methylation dispersion of each gene against chronological age.

Subsequently, a correlation analysis (Spearman test) was performed between each gene and age to determine if there is a correlation between methylation level and chronological age. Table 7 provides significant insights into the correlation between DNA methylation levels and chronological age for all three genes. The study of *ELOVL2* exhibits a strong positive association (r = 0.964) with chronological age, indicating a consistent increase in methylation as individuals age. *PRKG2* demonstrates a significant negative association (r = −0.955) between methylation levels and chronological age, indicating a decline in methylation as individuals become older. Similar to *PRKG2*, *EDARADD* exhibits a significant negative correlation (r = −0.942) with chronological age, suggesting a decrease in methylation levels as individuals age. The statistical significance (*p* < 0.001) of each gene emphasizes the strength of these relationships. Figure 4 shows the correlation between each gene and chronological age.

### 3.4. Predictive Model

We performed linear regression on each gene to obtain a prediction model. This prediction model is expected to be used in the future, where, when obtaining the DNA methylation status of an Indonesian, we can predict the person’s age by inputting the DNA methylation status into these formulas. After receiving the regression formula, we tested the MAE, R^2^, and RMSE as a matrix to determine the size of the prediction error and to determine the capability of the models to predict age.

**Figure 5:**
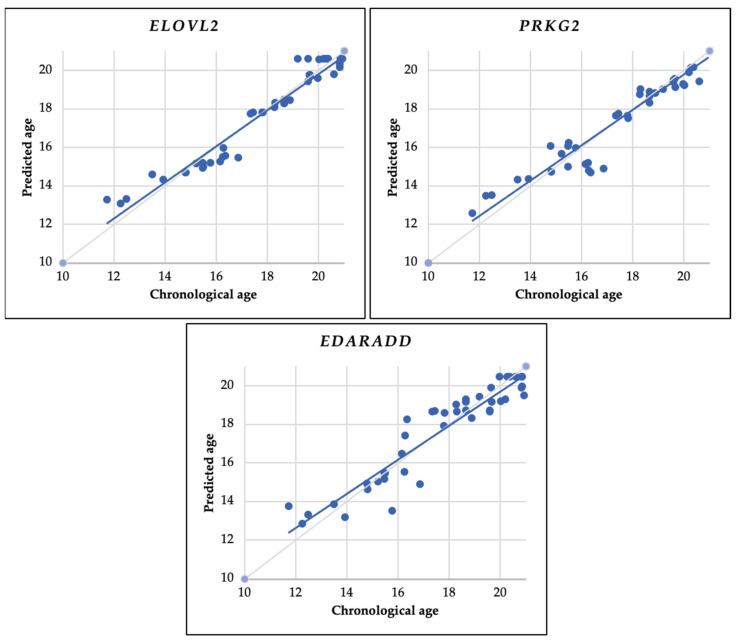
Using the entire sample (43 samples), this scatter plot illustrates the relationship between predicted age and chronological age. Predictions are based on linear regression analysis using the *ELOVL2*, *PRKG2*, and *EDARADD* genes. The continuous gray diagonal line represents perfect correlation, while the continuous blue diagonal line depicts the estimates in each model.

As shown in Table 8, the coefficient value (b) indicates the relationship of DNA methylation status with age, whereas, for the *ELOVL2* gene, the coefficient is positive, indicating a positive change in DNA methylation status with age. In contrast, for the *PRKG2* and *EDARADD* genes, there is a negative coefficient value, indicating a negative change in DNA methylation status with age. This result follows the Spearman test we performed earlier (Table 7). Meanwhile, the correlation between estimated and chronological age in the scatter plot is shown in Figure 5.

We used MAE, R^2^, and RMSE tests to evaluate the prediction model’s accuracy in estimating age. Our MAE and RMSE analysis determined that *ELOVL2* has the lowest value, while *EDARADD* has the highest value. Regarding the R^2^ test, the *ELOVL2* gene has the value closest to one, while the *EDARADD* gene has the value furthest from one (Table 8).

The multicollinearity test findings (Tolerance and VIF) indicate that all prediction models have a value of 1.000. This finding suggests no indication of multicollinearity in the prediction formulas (Table 8).

By following the formula Y = a + bX, where Y is age, a is a constant, b is the regression coefficient, and X is DNA methylation level, the prediction model for each gene can be written as follows:

Chronological Age = 12.215 + (0.084 ∗ *ELOVL2* Methylation Level) (1)

Chronological Age = 21.339 − (0.098 ∗ *PRKG2* Methylation Level) (2)

Chronological Age = 20.462 − (0.076 ∗ *EDARADD* Methylation Level) (3)

Subsequently, following the acquisition of the R^2^ values for each predictive model in Table 8, we performed a power analysis to determine if the sample size utilized in this research was sufficient to detect a significant effect if one exists. A higher power value corresponds to a superior ability of the prediction model to detect. Table 9 provides the power analysis calculation results for each gene we used as a predictor variable. We obtained 100% power for all genes. The prediction model generated in this research is highly probable to detect statistically significant effects based on the study parameters. The high power of our findings indicates that they are robust, and despite the small sample size, they are adequate for the analysis conducted in this study.

## 4. Discussion

Numerous CpG loci associated with age have been extensively documented within specific gene markers. This documentation facilitates age prediction through the analysis of DNA methylation levels. The underlying rationale of this analysis lies in the dynamic modulation of DNA methylation levels, which exhibit a discernible trajectory with advancing age. This modulation results from the intricate interplay between age-associated gene markers and environmental factors throughout an individual’s lifespan [16]. Conceptually, specific genes, such as *ELOVL2*, *NPTX2*, *FHL2*, *GRIA1*, *KCNAB3*, *PENK*, and *ASPA*, demonstrate an augmentation in DNA methylation, while others, including *PDE4C*, *PRKG2*, *EDARADD*, and *TOM1L1*, exhibit a decline [5,28,29,30]. According to Heyn et al., DNA methylation levels in CpG island regions manifest a predisposition toward hypermethylation. In contrast, regions with limited CpG sites, particularly those associated with tissue-specific genes, undergo hypomethylation. Generally, CpG sites in the elderly tend toward hypomethylation compared to the pediatric demographic [31]. This assertion supports the findings of McClay et al., who report that among 70 differentially methylated regions (DMRs), 42 undergo hypomethylation, while the remaining 28 exhibit hypermethylation concomitant with aging [32]. Similarly, Johansson et al. reveal that 60.5% experience hypomethylation with increasing age, while 39.5% undergo hypermethylation [33]. Additional insights were provided by Acevedo et al., showing that as children age, 41.6% undergo DNA methylation, while 58.4% experience DNA demethylation. Notably, with advancing age, demethylation occurs in regions of transposable elements and exons. Meanwhile, DNA methylation becomes evident in specific regions, including CpG islands, polycomb group (PcG) target genes, and promoters with bivalent chromatin domains [34,35]. Furthermore, the alterations in children’s DNA methylation patterns are approximately three to four times more diverse than in adults [21].

This study reveals a significant association between the *ELOVL2* (elongation of very long chain fatty acids), *PRKG2* (protein kinase, cGMP-dependent, Type II), and *EDARADD* (ectodysplasin A receptor-associated death domain) genes and the age of adolescents in the Indonesian population, specifically between the ages of 11 and 20 years. There was a significant positive correlation between the methylation status and the increase in age within the specified range in the *ELOVL2* gene with a correlation coefficient (r-value) of 0.96. Meanwhile, in contrast to *ELOVL2*, we observed a significant negative correlation between the DNA methylation levels of *PRKG2* and *EDARADD* genes and age, with correlation coefficients (R-values) of −0.95 and −0.94, respectively. The *ELOVL2* correlation coefficient is considered the highest among the other two genes, and it indicates that the gene has the strongest association between the ages of 11 and 20 years and the methylation level. This finding is congruent with the results of previous studies that *ELOVL2* is one of the genes that strongly correlate with age [14,36,37].

Furthermore, we performed linear regression analysis to determine the linear correlation between the independent and dependent variables and to generate prediction formulas that can be used for clinical age estimation scenarios. We obtained three prediction formulas from the three genes we used in this study. As a result, the three genes all had a *p*-value of 0.00 and 95% CI values within the same domain, which indicates a statistically significant correlation between each gene and chronological age.

Subsequently, to analyze the capability of the predictive formula, we used MAE, R^2^, and RMSE matrices. For MAE and RMSE values, the smaller the value of these two matrices, the better the formula. These values indicate that the predicted age produced by the formula is not much different from the chronological age. We found that the MAE and RMSE values of the *ELOVL2* prediction formula are the lowest, which are ±0.48 and ±0.38, respectively, compared to *PRKG2* (MAE: ±0.58, RMSE: ±0.55) and *EDARADD* (MAE: ±0.72, RMSE: ±0.79). Meanwhile, in contrast to the interpretation of MAE and RMSE results, in the R^2^ test, the higher the R^2^ value, the better. A number around one signifies the model’s capacity to elucidate a significant portion of the variability in the data. In contrast, if R^2^ is close to zero, the formula’s ability to explain the variability in the data is unfavorable. Therefore, when the R^2^ value approaches one, its quality improves. From the results of this test, we found that the R^2^ value of *ELOVL2* is the highest, measuring 0.94, followed by *PRKG2* and *EDARADD*, with values of 0.92 and 0.88, respectively. Therefore, the prediction formula generated by *ELOVL2* best explains the variability in the data.

Based on all the analysis tests we conducted, *ELOVL2* is considerably the most reliable indicator of age within this population age range. This finding aligns with the study conducted by Freire-Aradas et al., which concluded that *ELOVL2* has the highest correlation with age and can serve as the primary predictor in DNA methylation studies during the early stages of life [16]. This finding further enhances the previous research that has demonstrated the solid predictive capability of *ELOVL2* in determining DNA methylation levels in adults [14,36,38]. Moreover, the result of this research regarding the DNA methylation level in *PRKG2* and *EDARADD* aligns with the research conducted by Freire-Aradas et al., which indicated that those two genes exhibit hypomethylation in a young age range [16].

*ELOVL2* is involved in elongating LC-PUFAs (polyunsaturated fatty acids), specifically omega-3 and omega-6 fatty acids. Furthermore, this gene also codes for a transmembrane protein known as VLC-PUFAs (very long polyunsaturated fatty acids) and aids in the synthesis of DHA (docosahexaenoic acid), a specific type of omega-3 fatty acid consisting of 22 carbon atoms and six double bonds (22:6n-3). VLC-PUFAs have a significant impact on various particular tissues, including the nervous system development, maintenance of photoreceptor function and health, testes and spermatozoa development, and promotion of neuronal survival through paracrine signals with its metabolite (elovanoid) [39]. Meanwhile, DHA has been found to impact multiple aspects of health significantly. It helps promote heart health by lowering triglyceride levels and increasing HDL cholesterol. DHA also enhances brain function, particularly during pregnancy and early childhood. Adequate amounts of DHA are crucial for maintaining good eye health.

Additionally, DHA plays a vital role in reducing the intensity of inflammation during the inflammatory process. The role of fatty acids produced by *ELOVL2* highlights the essentiality of *ELOVL2* expression in early life for producing VLC-PUFAs and DHA. As we age, the ability of *ELOVL2* to generate fatty acids can be influenced by epigenetic factors, including DNA methylation. An elevation in DNA methylation within the regulatory region of this gene leads to a concomitant reduction in the regulation of *ELOVL2* expression at both the mRNA and protein levels. Consequently, there is a corresponding decline in the levels of VLC-PUFAs and DHA following the diminished regulation of *ELOVL2* expression. If there is an aberrant elevation in the degree of DNA methylation in this gene, it will interfere with lipid synthesis, resulting in heightened endoplasmic reticulum stress and mitochondrial malfunction. These modifications can accelerate the aging process, occurring at the molecular, physiological, or even phenotypic levels [14,36,39,40].

*PRKG2* codes for a serine or threonine protein kinase family protein. The protein exhibits diverse roles, including regulating intestinal secretion, bone growth and development, control of blood pressure through the regulation of renin secretion, regulation of endochondral ossification, and involvement in the proliferation of nerve cells and gastrointestinal cells. The precise cause for the decrease in the DNA methylation pattern of this gene with age is still unknown. Nevertheless, there could be a correlation between the function of the protein synthesized by this gene. Demethylation of *PRKG2* promotes intestinal secretion, regulates blood pressure, and supports the regeneration of nerve and gastrointestinal cells during the aging process [41].

*EDARADD* codes for a protein known as EDAR-associated protein via the death domain. This protein primarily functions during the prenatal period, facilitating the connection between the ectoderm and mesoderm embryonic cell layers and contributing to developing ectodermal features, such as hair, sweat glands, and teeth. Furthermore, it contributes to cellular activity by aiding the ectodysplasin A receptor in initiating chemical impulses within the cell. Additionally, it is involved in the immunological response. Subsequently, these signals will impact other cellular processes, including cell division, growth, and maturation. The precise mechanism behind the age-related decrease in DNA methylation levels in *EDARADD* has to be fully elucidated. Demethylation is believed to occur to promote the production of ectodermal structures, sustain cellular activity, and foster the development of the body’s immune system [42].

Creating the prediction models would enhance the clinical application of DNA methylation status analysis results in Indonesia. When forensic experts acquire a sample’s DNA methylation status, they can use this information as input for a prediction model to estimate the individual’s biological age. These prediction models offer a higher accuracy level (MAE) than earlier reports of age estimation using DNA methylation, which typically falls within 3 to 7 years [1,4,5,6,17,26,42,43]. With this excellent level of prediction accuracy, the prediction formula produced in this study can potentially be used to enforce age estimation in Indonesia relating to legal applications in the age range of 11–20 years.

However, despite achieving a highly satisfactory MAE, R^2^, and RMSE value, it is essential to gather more samples in future research to validate these findings and to increase the generalizability, reliability, and robustness. The process of sampling was a significant challenge in this research. During the COVID-19 pandemic, we could not directly obtain primary samples from patients. Therefore, we collected samples using an alternative method: obtaining blood samples from the RSPAD Clinical Pathology Laboratory data repository. In this scenario, confirming abnormal DNA methylation status was challenging because we could not establish environmental exposures or systemic disorders that might impact it. Even though we were able to retrieve the medical records of all patients, we were unable to track the diagnoses of any pre-existing systemic disorders prior to their arrival at RSPAD. For this investigation, we examined approximately 45,000 additional blood samples. However, only 43 samples satisfied the specified criteria for inclusion and exclusion because most patients who visit the Clinical Pathology Laboratory of RSPAD have medical conditions such as systemic lupus erythematosus, diabetes mellitus, aplastic anemia, leukemia, and cardiovascular ailments. We omitted these samples because prior research established a correlation between these disorders and irregularities in the DNA methylation state [40,44,45].

Furthermore, we discovered that specific samples exhibited insufficient DNA information after DNA extraction due to DNA degradation that occurs during the storage or transportation of blood samples. Therefore, we highly advise conducting future research by obtaining direct blood samples to verify environmental exposure, detect systemic abnormalities, and enhance control over the quality and quantity of collected samples. In addition, we strongly recommend that future DNA methylation studies cover a broader spectrum of DNA methylation patterns on more genes.

## 5. Conclusions

This study demonstrates a significant correlation between DNA methylation in the *ELOVL2*, *PRKG2*, and *EDARADD* genes and the chronological age of adolescents in the Indonesian population. More precisely, the methylation level of the *ELOVL2* gene has a robust positive association with age, making it a dependable marker for determining biological age within the 11–20-year-old age group. Conversely, the methylation levels of the *PRKG2* and *EDARADD* genes display a negative correlation with age, suggesting a decline in methylation levels as individuals age.

The study constructed a prediction model for each of the DNA methylation statuses of *ELOVL2*, *PRKG2*, and *EDARADD*. Based on the matrices employed (MAE, RMSE, and R^2^), the analysis suggests that the predictive model for the *ELOVL2* gene demonstrates more favorable results, as evidenced by the lowest MAE and RMSE values and the closest R^2^ value to one, compared to the other two genes. These prediction models can potentially enhance the practical application of DNA methylation investigations in Indonesia. Nevertheless, forthcoming studies about DNA methylation in Indonesia must incorporate a more extensive sample size to validate these findings and enhance the model’s precision.

Although the study yielded encouraging outcomes, it encountered various obstacles, such as the restricted sample size caused by the COVID-19 pandemic and the necessity to utilize secondary blood samples from the RSPAD Clinical Pathology Laboratory data collection. This circumstance makes it difficult to obtain a valid healthy sample because most patients visiting this laboratory had underlying systemic disorders, which are usually correlated with blood disorders and can affect DNA methylation levels in a person.

To improve the accuracy of DNA methylation status in samples, using primary blood samples directly from patients is recommended, allowing for better control over environmental exposures and systemic diseases. Moreover, analyzing the DNA methylation level on more genes may give us a broader spectrum of methylation patterns in the human body through aging.

## Figures and Tables

**Table 1:** Primer gene of *ELOVL2*, *PRKG2*, and *EDARADD.*

Gene	Sequence	Methylated (5′-3′)	Unmethylated (5′-3′)
*ELOVL2*	Forward	TTAGGGTTCGAATTTTAGAATTITC	AGGGTTTGAATTTTAGAATTTTTGA
	Reverse	CCCTATTACATACACGAAACCG	ATCCCCTATTACATACACAAAACCA
*PRKG2*	Forward	TTATTTGGAGAGGTTTGAAGC	TATTTGGAGAGGTTTGAAGT
	Reverse	TACTAAAATCCAAAAACGAAA	AAAAATACTAAAATCCAAAAACAAAA
*EDARADD*	Forward	CGTAGGTAAAGGGATATAGCGTC	TGTAGGTAAAGGGATATAGTGTTG
	Reverse	CTAAAAACCCCGTACGACTCC	AAACTAAAAACCCCATACAACTCC

**Table 2:** Characteristics of research respondents based on chronological age (n = 43).

Characteristic	Mean ± S.D ^a^	S.E. Mean ^b^	Median	Mode	Minimum	Maximum
Age (year)	17.62 ± 2.60	0.40	18.29	18.65	11.73	20.93

^a^ S.D. = standard deviation; ^b^ S.E. Mean = standard error of mean.

**Table 3:** Kappa test analysis results between two observers.

Measurement of Agreement	Value	Asymptomatic Standard Error ^a^	Approximate T ^b^	Approximate Significance
Kappa	0.909	0.021	20.573	0.000
N of Valid Case	172			

^a^ Not assuming the null hypothesis. ^b^ Using the asymptomatic standard error assuming the null hypothesis.

**Table 4:** Description of methylation status in the center size table.

Genes	Methylation Level (%)	Mean ± S.D ^a^	S.E. Mean ^b^	Median	Mode	Minimum	Maximum
*ELOVL2*	Methylated	64.16 ± 29.99	4.57	70.02	100.00	10.40	100.00
	Unmethylated	35.84 ± 29.99	4.57	29.98	0.00	0.00	89.60
*PRKG2*	Methylated	38.00 ± 25.54	3.90	30.83	0.00	0.00	89.23
	Unmethylated	62.00 ± 25.54	3.90	69.17	100.00	10.77	100.00
*EDARADD*	Methylated	37.45 ± 32.30	4.93	23.51	0.00	0.00	99.83
	Unmethylated	62.55 ± 32.30	4.93	76.49	100.00	0.17	100.00

^a^ S.D. = standard deviation; ^b^ S.E. Mean = standard error of mean.

**Table 5:** Kruskal–Wallis test analysis results for methylation level.

	Median (Min–Max)	*p*
*ELOVL2*	70.02 (10.40–100.00)	<0.001 *
*PRKG2*	30.83 (0.00–89.23)	
*EDARADD*	23.51 (0.00–99.83)	

* *p* < 0.05.

**Table 6:** Significance of the Mann–Whitney post-hoc test analysis results.

	*ELOVL2*	*EDARADD*
*PRKG2*	<0.001 *	0.656
*ELOVL2*		<0.001 *

* *p* < 0.05.

**Table 7:** Correlation analysis between each gene and chronological age.

Genes	Methylation Level Median (Min–Max)	Chronological Age	*r*	*p*	CI ^a^
*ELOVL2*	70.02 (10.40–100.00)	17.62 ± 2.60	0.964 **	<0.001	0.020 to 0.066
*PRKG2*	30.83 (0.00–89.23)	17.62 ± 2.60	−0.955 **	<0.001	−0.054 to −0.008
*EDARADD*	23.51 (0.00–99.83)	17.62 ± 2.60	−0.942 **	<0.001	−0.033 to −0.002

^a^ CI = Confidence Interval; ** *p* < 0.01.

**Table 8:** Predictive model for each gene.

Gene	Constant (a)	Coefficient (b)	*p*	95% CI	MAE ^a^	R^2^	RMSE ^b^	Multicollinearity	
								Tolerance	VIF ^c^
*ELOVL2*	12.215	0.084	0.000 **	0.078 to 0.091	±0.48	0.94	±0.38	1.000	1.000
*PRKG2*	21.339	−0.098	0.000 **	−0.107 to −0.088	±0.58	0.92	±0.55	1.000	1.000
*EDARADD*	20.462	−0.076	0.000 **	−0.084 to −0.067	±0.72	0.88	±0.79	1.000	1.000

** *p* < 0.01; ^a^ MAE = mean absolute error; ^b^ RMSE = root mean squared error; ^c^ VIF = variance inflation factor.

**Table 9:** Power analysis in each gene.

Genes	R^2^	Power (%)	Significance Level
*ELOVL2*	0.94	100	0.05
*PRKG2*	0.92	100	0.05
*EDARADD*	0.88	100	0.05

## Data Availability

The original contributions presented in the study are included in the article; further inquiries can be directed to the corresponding author.

## References

[1] Freire-Aradas A., Phillips C., Lareu M.V. (2017). Forensic individual age estimation with DNA: From initial approaches to methylation tests. Forensic Sci. Rev..

[2] Schmeling A., Dettmeyer R., Rudolf E., Vieth V., Geserick G. (2016). Forensic Age Estimation: Methods, Certainty, and the Law. Dtsch. Aerzteblatt Online.

[3] Jia L., Zhang W., Chen X. (2017). Common methods of biological age estimation. Clin. Interv. Aging.

[4] Meng H., Ma K.J., Dong L.M., Li C.T., Xiao B., Xu L.Y., Huang P., Xie J.H. (2019). Research Progress on Age Estimation Based on DNA Methylation. J. Forensic Med..

[5] Hanafi M.S., Soedarsono N., Auerkari E. (2021). Biological age estimation using DNA methylation analysis: A systematic review. Sci. Dent. J..

[6] Maulani C., Auerkari E.I. (2020). Age estimation using DNA methylation technique in forensics: A systematic review. Egypt. J. Forensic Sci..

[7] Luciana M., Bjork J.M., Nagel B.J., Barch D.M., Gonzalez R., Nixon S.J., Banich M.T. (2018). Adolescent neurocognitive development and impacts of substance use: Overview of the adolescent brain cognitive development (ABCD) baseline neurocognition battery. Dev. Cogn. Neurosci..

[8] Liu Y.-Y., Harbison S. (2018). A review of bioinformatic methods for forensic DNA analyses. Forensic Sci. Int. Genet..

[9] Sen P., Shah P.P., Nativio R., Berger S.L. (2016). Epigenetic Mechanisms of Longevity and Aging. Cell.

[10] Horvath S., Raj K. (2018). DNA methylation-based biomarkers and the epigenetic clock theory of ageing. Nat. Rev. Genet..

[11] Jung M., Pfeifer G.P. (2015). Aging and DNA methylation. BMC Biol..

[12] Richards R., Patel J., Stevenson K., Harbison S. (2019). Assessment of DNA methylation markers for forensic applications. Aust. J. Forensic Sci..

[13] Eipel M., Mayer F., Arent T., Ferreira M.R.P., Birkhofer C., Gerstenmaier U., Costa I.G., Ritz-Timme S., Wagner W. (2016). Epigenetic age predictions based on buccal swabs are more precise in combination with cell type-specific DNA methylation signatures. Aging.

[14] Spólnicka M., Pośpiech E., Pepłońska B., Zbieć-Piekarska R., Makowska Ż., Pięta A., Karłowska-Pik J., Ziemkiewicz B., Wężyk M., Gasperowicz P. (2018). DNA methylation in ELOVL2 and C1orf132 correctly predicted chronological age of individuals from three disease groups. Int. J. Leg. Med..

[15] Parson W. (2018). Age Estimation with DNA: From Forensic DNA Fingerprinting to Forensic (Epi)Genomics: A Mini-Review. Gerontology.

[16] Freire-Aradas A., Phillips C., Girón-Santamaría L., Mosquera-Miguel A., Gómez-Tato A., Casares de Cal M.Á., Álvarez-Dios J., Lareu M.V. (2018). Tracking age-correlated DNA methylation markers in the young. Forensic Sci. Int. Genet..

[17] Correia Dias H., Cordeiro C., Corte Real F., Cunha E., Manco L. (2020). Age Estimation Based on DNA Methylation Using Blood Samples From Deceased Individuals. J. Forensic Sci..

[18] Horvath S., Gurven M., Levine M.E., Trumble B.C., Kaplan H., Allayee H., Ritz B.R., Chen B., Lu A.T., Rickabaugh T.M. (2016). An epigenetic clock analysis of race/ethnicity, sex, and coronary heart disease. Genome Biol..

[19] Aliferi A., Ballard D., Gallidabino M.D., Thurtle H., Barron L. (2018). Syndercombe Court D DNA methylation-based age prediction using massively parallel sequencing data and multiple machine learning models. Forensic Sci. Int. Genet..

[20] Gopalan S., Carja O., Fagny M., Patin E., Myrick J.W., McEwen L.M., Mah S.M., Kobor M.S., Froment A., Feldman M.W. (2017). Trends in DNA Methylation with Age Replicate Across. Genetics.

[21] Alisch R.S., Barwick B.G., Chopra P., Myrick L.K., Satten G.A., Conneely K.N., Warren S.T. (2012). Age-associated DNA methylation in pediatric populations. Genome Res..

[22] Wu X., Chen W., Lin F., Huang Q., Zhong J., Gao H., Song Y., Liang H. (2019). DNA methylation profile is a quantitative measure of biological aging in children. Aging.

[23] Breton C.V., Marsit C.J., Faustman E., Nadeau K., Goodrich J.M., Dolinoy D.C., Herbstman J., Holland N., LaSalle J.M., Schmidt R. (2017). Small-magnitude effect sizes in epigenetic end points are important in children’s environmental health studies: The children’s environmental health and disease prevention research center’s epigenetics working group. Environ. Health Perspect..

[24] Almstrup K., Lindhardt Johansen M., Busch A.S., Hagen C.P., Nielsen J.E., Petersen J.H., Juul A. (2016). Pubertal development in healthy children is mirrored by DNA methylation patterns in peripheral blood. Sci. Rep..

[25] Mayer F., Becker J., Reinauer C., Böhme P., Eickhoff S.B., Koop B., Gündüz T., Blum J., Wagner W., Ritz-Timme S. (2022). Altered DNA methylation at age-associated CpG sites in children with growth disorders: Impact on age estimation?. Int. J. Leg. Med..

[26] Shi L., Jiang F., Ouyang F., Zhang J., Wang Z., Shen X. (2018). DNA methylation markers in combination with skeletal and dental ages to improve age estimation in children. Forensic Sci. Int. Genet..

[27] Hartomo B.T. (2019). Pemeriksaan Biomolekuler Metilasi DNA untuk Prakiraan Usia pada Kelompok Anak-anak dan Dewasa. Master’s Thesis.

[28] Dhingra R., Kwee L.C., Diaz-Sanchez D., Devlin R.B., Cascio W., Hauser E.R., Gregory S., Shah S., Kraus W.E., Olden K. (2019). Evaluating DNA methylation age on the Illumina MethylationEPIC Bead Chip. PLoS ONE.

[29] Li S.-F., Peng F.-D., Wang J.-N., Zhong J.-J., Zhao H., Wang L., Li Y.-J., Liu F., Li C.-X. (2019). Methylation-Based Age Estimation Model Construction and Its Effectiveness Evaluation. J. Forensic Med..

[30] Hamano Y., Manabe S., Morimoto C., Fujimoto S., Ozeki M., Tamaki K. (2016). Forensic age prediction for dead or living samples by use of methylation-sensitive high resolution melting. Leg. Med..

[31] Heyn H., Li N., Ferreira H.J., Moran S., Pisano D.G., Gomez A., Diez J., Sanchez-Mut J.V., Setien F., Carmona F.J. (2012). Distinct DNA methylomes of newborns and centenarians. Proc. Natl. Acad. Sci. USA.

[32] McClay J.L., Aberg K.A., Clark S.L., Nerella S., Kumar G., Xie L.Y., Hudson A.D., Harada A., Hultman C.M., Magnusson P.K. (2014). A methylome-wide study of aging using massively parallel sequencing of the methyl-CpG-enriched genomic fraction from blood in over 700 subjects. Hum. Mol. Genet..

[33] Johansson Å., Enroth S., Gyllensten U. (2013). Continuous Aging of the Human DNA Methylome Throughout the Human Lifespan. PLoS ONE.

[34] Acevedo N., Reinius L.E., Vitezic M., Fortino V., Söderhäll C., Honkanen H., Veijola R., Simell O., Toppari J., Ilonen J. (2015). Age-associated DNA methylation changes in immune genes, histone modifiers and chromatin remodeling factors within 5 years after birth in human blood leukocytes. Clin. Epigenetics.

[35] Rakyan V.K., Down T.A., Maslau S., Andrew T., Yang T.P., Beyan H., Whittaker P., McCann O.T., Finer S., Valdes A.M. (2010). Human aging-associated DNA hypermethylation occurs preferentially at bivalent chromatin domains. Genome Res..

[36] El-Shishtawy N.M., El Marzouky F.M., El-Hagrasy H.A. (2024). DNA methylation of ELOVL2 gene as an epigenetic marker of age among Egyptian population. Egypt. J. Med. Human. Genet..

[37] Zbieć-Piekarska R., Spólnicka M., Kupiec T., Makowska Ż., Spas A., Parys-Proszek A., Kucharczyk K., Płoski R., Branicki W. (2015). Examination of DNA methylation status of the ELOVL2 marker may be useful for human age prediction in forensic science. Forensic Sci. Int. Genet..

[38] Bacalini M.G., Deelen J., Pirazzini C., De Cecco M., Giuliani C., Lanzarini C., Ravaioli F., Marasco E., Van Heemst D., Suchiman H.E.D. (2017). Systemic Age-Associated DNA Hypermethylation of ELOVL2 Gene: In Vivo and in Vitro Evidences of a Cell Replication Process. J. Gerontol.-Ser. A Biol. Sci. Med. Sci..

[39] Chao D.L. (2020). Skowronska-Krawczyk D ELOVL2: Not just a biomarker of aging. Transl. Med. Aging.

[40] Ramo-Fernández L., Karabatsiakis A., Boeck C., Bach A.M., Gumpp A.M., Mavioglu R.N., Ammerpohl O., Kolassa I.T. (2022). Characterization of the effects of age and childhood maltreatment on ELOVL2 DNA methylation. Dev. Psychopathol..

[41] Zhang M., Ali G., Komatsu S., Zhao R., Ji H.L. (2022). Prkg2 regulates alveolar type 2-mediated re-alveolarization. Stem Cell Res. Ther..

[42] Ogata A., Kondo M., Yoshikawa M., Okano M., Tsutsumi T., Aboshi H. (2022). Dental age estimation based on DNA methylation using real-time methylation-specific PCR. Forensic Sci. Int..

[43] Guan X., Ohuchi T., Hashiyada M., Funayama M. (2021). Age-related DNA methylation analysis for forensic age estimation using post-mortem blood samples from Japanese individuals. Leg. Med..

[44] Beygo J., Ammerpohl O., Gritzan D., Heitmann M., Rademacher K., Richter J., Caliebe A., Siebert R., Horsthemke B., Buiting K. (2013). Deep Bisulfite Sequencing of Aberrantly Methylated Loci in a Patient with Multiple Methylation Defects. PLoS ONE.

[45] Heyn H., Moran S., Esteller M. (2013). Aberrant DNA methylation profiles in the premature aging disorders Hutchinson-Gilford Progeria and Werner Syndrome. Epigenetics.

